# Lifestyle Choices and Brain Health

**DOI:** 10.3389/fmed.2019.00204

**Published:** 2019-10-04

**Authors:** Jacobo Mintzer, Keaveny Anne Donovan, Arianne Zokas Kindy, Sarah Lenz Lock, Lindsay R. Chura, Nicholas Barracca

**Affiliations:** ^1^Roper Saint Francis Research and Innovation Center, Charleston, SC, United States; ^2^Ralph H. Johnson VA Medical Center, Charleston, SC, United States; ^3^AARP, Washington, DC, United States; ^4^Global Council on Brain Health, Washington, DC, United States

**Keywords:** brain health, geriatrics, lifestyle, prevention, dementia, cognitive decline

## Abstract

This article is largely based on the recommendations of the AARP's Global Council on Brain Health (GCBH) and aims to provide an overview of evidence from current literature and expert opinion on key elements known to be relevant in preserving brain health as people age. Although we realize that there may be other lifestyle choices of importance to brain health, the GCBH has decided to initially focus on the issues below based on the preferences and concerns of its members. The areas to be discussed are: mental well-being, exercise, cognitively stimulating activities, sleep, nutrition, and social connectedness. Our review concluded that each of these areas offer opportunities for aging individuals to make lifestyle adjustments to positively impact brain health.

## Introduction

This article is largely based on the recommendations of the AARP's Global Council on Brain Health (GCBH) (1–6). The AARP is a United States-based interest group whose stated mission is “to empower people to choose how they live as they age”. According to the organization, it had more than 38 million members as of 2018 (7). The article aims to provide an overview of the evidence from current literature and expert opinion on key issues known to be relevant in preserving brain health as people age. The GCBH is an independent group of scientists, health professionals, scholars, and policy experts from around the world working in areas of brain health related to human cognition. The GCBH is convened by the AARP in collaboration with Age UK. It follows a structured process for generating reliable information, which includes conducting extensive literature reviews and holding GCBH member meetings to discuss lifestyle issue areas that may impact brain health as people age. The goal of the group is to provide specific, evidence-based recommendations for people to consider incorporating into their lives to maintain and improve brain health as they age. The issues discussed here reflect the priorities established by the GCBH. We acknowledge that other entities may disagree on the relevance of the topics presented here. The recommendations included within this review are related to mental well-being, exercise, cognitively stimulating activities, sleep, nutrition, and social connectedness. Professionals and consumers are constantly overwhelmed with contradictory information about changes in lifestyle that can impact brain health. The AARP created the GCBH to evaluate the evidence that emerges and provide the professional and the lay consumer with accurate and scientifically valid information to guide the lifestyle choices they make to improve their brain health. Therefore, the goal of this paper is to summarize these recommendations for professionals.

## Brain Health and Mental Well-Being

The goal of this section is to explore the relationship between mental well-being and brain health. Mental well-being, for the purpose of this article, is defined as experiences characterized by feeling good, functioning well, and adequately coping with life circumstances and challenges.

The GCBH's interest in this topic was triggered by the results of the 2018 AARP Brain Health and Mental Well-Being Survey. The survey was performed on 2,287 randomly selected, community dwelling adults in the USA. The subgroup age over 50 (*N* = 1,263) was analyzed to better understand the relationship between mental well-being and brain health in this group. It was found that people who scored higher on the scale of mental well-being tended to report better memory and thinking skills than those who reported lower mental well-being. Furthermore, the AARP found that when a subject rated their episodic memory and executive function as excellent or very good, they also reported very high rates of mental well-being. Comparatively, those who rated the same functions as average, fair, or poor reported low rates of mental well-being. Interestingly, the largest difference shown was in how people rated their mental sharpness. Those who rated themselves as having low mental sharpness reported a nearly 17-point difference (38.8 compared to 55.5) in mental well-being from those who said their mental sharpness was excellent or very good (8). Based on the results of this survey, we hypothesize that there is a positive correlation between mental well-being and brain health.

Our literature search shows many scientific studies that explore the same hypothesis. For example, Sutin, Stephan, and Terracciano (9) evaluated the roles of optimism and life purpose in brain health. They found that greater optimism, positive attitude, positive affect, life satisfaction, and purpose in life were associated with reduced risk of dementia. When this data was combined with socioeconomic, psychological, and generational variables, they discovered that a sense of a purpose in life was one of the strongest predictors of better brain health. Furthermore, the study found that having a purpose in life was associated with a 20% reduction in dementia risk. An additional study conducted by Galderisi et al. corroborated these findings and suggested that a meaningful and goal-driven life also reduces the risk of dementia (10).

If it's agreed that mental well-being contributes to brain health, then we should explore which lifestyle choices have an effect on mental well-being. For example, as we discuss later in the section titled “The Brain and Social Connectedness,” individuals with strong community links have been associated with both mental well-being and better brain health when compared with the control group (11, 12). The same is true for exercise, which is addressed in “The Brain-Body Connection” portion of this review (13–21).

The opposite also appears to be true. Research has shown that early-life stress influences mental well-being and the brain. Significant adverse events early in life can render people vulnerable to mental illness later in life (22). Even during the time of gestation, exposure to excessive levels of cortisone resulting from high levels of stress can have long lasting effects by influencing brain development, subsequently lowering future levels of mental well-being (23–27). For example, researchers have specifically evaluated the effects of early-life adversity (28–32). The research showed a reduction of brain regions responsible for cognition, such as the hippocampus, in individuals that were exposed to early-life adversity (33, 34). Furthermore, the potential relationship between negative early-life experiences and the presence of amyloid (the basic lesion observed in Alzheimer's Disease) has been suggested in several animal studies including mouse models of Alzheimer's Disease (35).

Research shows that as we age, we are exposed to both physical and emotional losses. However, despite the increase in incidences associated with loss, getting older does not necessarily mean experiencing less happiness. People who manage stress effectively most or all of the time report greater well-being as they pass through later stages of life after the age of 50 (36). In addition, it should be considered that the way that individuals successfully manage stress is affected by cultural perceptions, social-economic status, family structure, and environment (11, 37, 38).

A final pertinent factor to consider is the relationship between severe depression and dementia risk. It has been discovered that individuals who have normal memory but suffer from severe depression are likely to develop dementia within a few years (39). Memory loss symptoms caused by Alzheimer's and other related diseases may not manifest themselves for up to 15–20 years after the disease has begun to develop (40). Because of this, it is important to acknowledge the possibility that late-life depression could be the only early manifestation of Alzheimer's disease in a given individual.

In summary, research shows that mental well-being appears to be strongly associated with brain health and could potentially be a protective factor in the onset of neurodegenerative disorders.

## The Brain-Body Connection

For many years, clinicians have recommended physical activity under the belief that it will have a positive impact on brain health no matter if it results from having a physically active lifestyle (walking to work or the store in place of driving, taking the stairs, and engaging in hobbies and sports) or from purposeful exercise (brisk walking, strength training, and aerobic training).

Current research strongly supports this belief. By analyzing animal studies, scientists were able to determine that exercise facilitates neuroplasticity and improves learning outcomes (17, 18). The same seems to be true for humans. Coelho found that physical exercise effectively increases the peripheral levels of brain-derived neurotrophic factor (BDNF), a protein in the brain that promotes growth and maintenance of neurons in the elderly (15). Furthermore, Baker et al. conducted a randomized trial comparing both high-intensity aerobic exercise and stretching to stretching alone over 6 months (14). The results showed the sex-specific effects of aerobic exercise on cognition, glucose metabolism, and hypothalamic-pituitary-adrenal axis and trophic activity as well as comparable improvements in cardiorespiratory fitness and body fat reduction. Interestingly, different results were observed in women and men. For women, aerobic exercise enhanced performance on multiple tests. Their results showed improved executive function while increasing glucose disposal during the metabolic clamp and reducing fasting plasma levels of insulin, cortisol, and BDNF. For men, aerobic exercise increased plasma levels of insulin-like growth factor 1, but only had a favorable effect on a single measure of performance (17).

Other studies have focused on demonstrating the neurological correlations between aerobic exercise and brain health (41). For example, a study by Colcombe et al. examined whether aerobic fitness training in older adults can increase brain volume in regions associated with age-related decline in both brain structure and cognition. They found a significant increase in both gray and white matter regions of the brain in those who participated in the aerobic fitness training but not in the older adults who participated in the stretching and toning (non-aerobic) control group (17). Finally, several meta-analyses have confirmed the role of exercise in brain health. These studies documented a significantly reduced risk of dementia and mild cognitive impairment associated with midlife exercise (13, 16, 18–21, 42–45).

While not all studies relating physical activity to brain heath have showed corresponding results (46), the GCBH felt that the evidence was strong enough to support a recommendation consistent with those from the American Heart Association of 150 min of moderate-intensity aerobic exercise and 2 or more days a week of moderate-intensity weight training (47).

## Cognitively Stimulating Activities

Cognitively stimulating activities are defined as mentally engaging activities or exercises that challenge a person's ability to think and process information. These include mind-teaser games, educational activities, intellectual inquiries, and mental challenges.

It has been well-established that brain structures and functions change as an individual ages (48). These changes, however, are not universal. Some individuals at the age of 80 have the same volume of certain brain structures associated with cognitive function, such as the hippocampus, as they did in their 30s. Others show substantial volume loss (49). The challenge is to understand which factors are responsible for these important differences. Education and learning have been known to enhance cognitive reserves, making an individual less susceptible to the effects of age or disease-related brain changes (50).

Research has shown that brain plasticity continues to be present through the aging process; therefore, we can hypothesize that cognitively stimulating activities could help maintain cognitive function or delay cognitive decline (51, 52). Supporting this hypothesis, several studies have established a link between participation in self-initiated, cognitively stimulating activities and brain health (53–59).

Unfortunately, this hypothesis still has yet to be demonstrated. The National Academies committee concluded that the “AHRQ (U.S. Agency for Healthcare Research and Quality) systematic review identified no specific interventions to justify mounting an assertive public health campaign to encourage people to adopt them for the purpose of preventing cognitive decline and dementia” (60). This includes performing what can be described as cognitively stimulating activities.

In conclusion, although there is a large body of evidence suggesting that cognitive training can improve reasoning, memory, and speed of processing, the validity of these connections has yet to be documented in large randomized control trials. It is important to note that no specific “brain game” or “brain exercise” has shown to be effective on brain health.

## The Brain-Sleep Connection

Sleep patterns change during the aging process, with both the sleep structure and duration becoming significantly altered (61). Even though sleep patterns vary substantially between age 25 and 50, the need for sleep does not differ. Recent literature has shown that most adults require between 7 and 8 h of sleep a night to main good physical and brain health (62). Some of the major changes observed are related to depth of sleep and continuity of sleeping patterns. While most older adults experience no changes in the length of time it takes them to fall asleep, interruption of sleep during the night and early wakening are common. Additionally, older adults also tend to wake up earlier in the morning and find that staying up late becomes more difficult. These changes are a normal part of the aging process and do not affect brain health. However, decreased total sleep time does indeed cause negative effects (9, 63–65).

In the AARP 2016 Sleep and Brain Health Survey, it was found that 44% of adults 50 years of age or older rated their quality of sleep as extremely good or very good; 84% said they were mostly well-rested in the morning; but only 33% reported being very satisfied with the amount of sleep they got daily (65). The 2016 AARP survey also discovered that while over half (56%) of adults 50 and older reported that they wake up at about the same time every morning, less than a half (48%) go to bed at same time and only a third (33%) report getting 7–8 h of sleep at night. Researchers have found that attention memory and executive function can be negatively affected by getting <8 h of sleep (66, 67), emphasizing the importance of consistent sleep patterns.

Shifts in normal patterns of sleep aside, lack of exposure to light can cause chemical imbalances, thus altering sleep patterns (68). Additional factors that can disrupt sleep are medical regimens designed to treat a variety of chronic diseases and debilitating sleep disorders that become more prevalent as individuals age. Fortunately, several lifestyle measures can be taken to overcome these negative shifts. Increasing time in the sunlight, consistent sleep routines, and gaining knowledge about the effects of medications are all vital components in preserving sleep patterns (69–72).

Light exposure has a large influence on the chemical balance involved in sleep maintenance. Exposure to light is critical to maintaining adequate secretion of melatonin (68, 70). The systematic secretion of neurochemicals such as melatonin provides a key regulatory process for other brain chemicals to follow in maintaining sleep patterns. Research has shown that older adults often have limited exposure to daylight. Because of this, studies suggest spending time outside without wearing sunglasses and allowing for higher exposure of light during the daytime to improve sleep patterns (73). The use of indoor light enhancement like phototherapy lamps can also improve sleep and cognition in elderly adults (74, 75).

Secondly, multi-medicine regimens in aging individuals can be very concerning and have the potential to alter healthy sleep patterns. Although a multi-medicine regimen is often needed to manage chronic disorders, specialists should be aware of the negative effects benzodiazepines, anticholinergics, hypnotics, barbiturates, antipsychotics, some antidepressants, and prescribed or over-the-counter (OTC) medicines have on older adults (76). It is important for clinicians to remember that many OTC medicines have mixtures of compounds that can impair sleep (77). Patients should also be encouraged to gather more information from their health care providers to learn about the potential negative effects multi-medicine regimens have on their sleep patterns.

In regards to pathological conditions related to sleep, insomnia is the most widely reported sleep disorder in older adults (78). Insomnia in older adults is often inaccurately characterized as difficulty falling asleep when it is actually fragmented sleep and difficulty staying asleep. Large studies have clearly documented the risk fragmented sleep poses to brain health (79). Older adults who have fragmented sleep vs. those who do not are at an increased risk of cerebral small vessel disease and poor cognitive and emotional functioning (66). Furthermore, insomnia carries an increased risk factor for stroke and is one of the main contributors to the development of depression (80, 81). Approaches to treat insomnia include behavioral and lifestyle changes, limiting the need for pharmaceutical treatment to induce and sustain sleep (82).

Sleep apnea is also very common sleep disorder in older adults. It is characterized by the collapsing of airways during sleep, thus making it difficult to breath. Approximately one-third of individuals suffering from sleep apnea experience severe symptoms, while up to two-thirds of adults 65 years and older will experience symptoms ranging from mild to severe (83). Sleep apnea has been shown to cause degeneration of brain tissue and the brain stem (84, 85). Fortunately, research has shown that some of the damage can be reversed when appropriate treatment is implemented, improving sleep quality and increasing blood-oxygen levels (63). The tissues in our airways begin to sag as we age, causing the airway to collapse and consequently resulting in sleep apnea. Furthermore, individuals aged 65 and older suffer from increased rates of cognitive impairment and dementias when ailed with sleep apnea. Studies prove, however, that improvements in cognitive function are possible in Alzheimer's patients with sleep apnea when treated with continuous positive airways pressure (CPAP) (86).

Experts will agree that one key element to maintaining brain health is an adult's ability to sleep 7–8 h in a 24-h period. According the 2016 AARP survey, 45% of adults 55 years and older in the USA nap once a week or more (9). It is possible for an individual to complete their 7–8 h of necessary sleep with regular napping; however, this concept is controversial (87). Despite some studies suggesting that naps may be useful for older adults, there have not been large studies performed to test the effects of napping on cognition. It is important to note that naps of 30 min or less in the early afternoon have not resulted in any negative effects on nighttime sleep, but long, late afternoon naps can upset nighttime sleep and generate disruptions in the sleep cycle.

Finally, changes in hormones observed in women's transition through perimenopause and menopause can cause sleep disturbances (88). Surges of adrenaline often result in hot flashes that can cause stress and changes in body temperature, resulting in the disruption of sleep patterns (89).

In summary, obtaining 7–8 h of sleep per 24-h period has been reported to be critical in preserving brain health. Paying special attention to lifestyle habits such as a routine sleep pattern as well as monitoring prescribed and OTC medicines and total length of exposure to light can be extremely beneficial. Napping in the early afternoon can also be productive if it is utilized to complete the 7 to 8-h recommended sleep duration. Lastly, sleep disorders such as sleep apnea and insomnia are common with age so it is vital to identify and treat them before they cause permanent disruption in sleep patterns.

## Brain Food

The GCBH also examined the link between certain nutrients, foods, and dietary patterns that could potentially lead to better cognitive outcomes in older adults (90–92). To that end, this review considered studies including nutritional mechanisms, observational studies, and randomized controlled trials (93–103). These studies have shown that eating patterns affect brain health over an individual's entire life span, supporting the importance of eating a balanced diet. For example, there is growing evidence that nutrients such as vitamins and minerals are highly beneficial when taken as part of a balanced diet rather than as concentrated supplements. The following are various diets that were evaluated by the GCBH and determined to be “brain-healthy”:

### The Mediterranean Diet

The Mediterranean Diet [MeDi] reflects the patterns of food consumption in Mediterranean countries such as Greece, Italy, and Spain. In general terms, it is characterized by a high intake of monounsaturated fats (with extra virgin olive oil as the main source), vegetables, fruits, plant proteins, whole grains, and fish. This diet limits the consumption of red meat, refined grains, and sweets. It often includes the moderate intake of wine, usually red, during one of the meals. Numerous studies, observational, and controlled, (93–97) have found that the MeDi provides additional health benefits such as decreased risk of AD, cardiovascular disease, and type two diabetes. For example, a study conducted by Scarmeas et al. evaluated the effects of the MeDi on over 2,000 New York residents over a 1.5-year period. The results showed that subjects who moderately adhered to the MeDi experienced 15–21% less risk for development of AD, whereas those who strongly adhered to the MeDi had 39–40% less risk for development of AD (93).

### Nordic Diet

The Nordic Diet draws upon the types of food consumed in Scandinavian countries including Denmark, Finland, Iceland, Norway, and Sweden (98). As in the case of the MeDi, there is an emphasis on non-animal nutrients such as fruits and vegetables. The Nordic Diet has also incorporated the consumption of fish, oils, and several types of meat. However, the MeDi and the Nordic Diet diverge in regards to the specific vegetables, fruits, cooking styles, and the type and quantity of oil utilized. For example, the Nordic Diet uses rapeseed oil (canola oil) instead of olive oil. A 4-year study on 1,140 randomly selected men and women with normal cognition was conducted to examine the cross-sectional and longitudinal associations of the Nordic Diet with cognitive function. Assessments by the Consortium to Establish a Registry for Alzheimer's Disease (CERAD) neuropsychological battery and the Mini-mental State Examination (MMSE) showed that participants who followed the guidelines of the Nordic Diet experienced increased levels of cognitive functioning compared to baseline (99).

### DASH (Dietary Approaches to Stop Hypertension)

The DASH Diet is characterized by a low intake of sodium and small portion sizes, which studies have shown to have substantial health benefits. These include improved blood pressure and blood lipids as well as a reduced risk of chronic disease (100). The difference between the DASH Diet vs. the MeDi and Nordic diet is the DASH Diet's inclusion of nuts, low-fat and non-fat dairy products, lean meats, and whole grains. A study conducted by Appel et al. included 459 adults with systolic blood pressures of <160 mm Hg and diastolic blood pressures of 80–95 mm Hg, 133 of which suffered from hypertension. After 3 weeks of adhering to the DASH Diet, the 133 subjects with hypertension decreased their systolic and diastolic blood pressure by 11.4 and 5.5 mm Hg more, respectively, than those in the control group (101).

### Okinawan Diet

The Okinawan diet is based on the eating habits of indigenous people residing in the Ryukyu Islands of Japan. Their eating habits were studied as scientists became aware of the population's exceptional longevity. The Okinawan diet emphasizes the consumption of yellow, orange, and green vegetables and includes soy and legumes. It also limits the intake of refined grains, sugar, salt, and diary. Notably, the diet focuses on the use of sweet potato and very small quantities of fish and rice. An additional point of interest is that this group does not eat until feeling full, but until hunger has subsided. A study done by Miyagi et al. discovered that, in 1990, the residents of Okinawa's life expectancy at birth (LEB) was of the highest in the world while their risk of ischemic heart disease and cerebrovascular disease was at a record low. The evidence showed that their superior health was due to a daily intake of meat and pulses at 90 grams, which is about 20 and 30% higher than the national average, respectively. Another contributor to their notable LEB and decreased risk of disease was their daily intake of green and yellow vegetables was about 50% higher than the national average (102).

### MIND (Mediterranean-DASH Intervention for Neurodegenerative Delay) Diet

The MIND diet draws from both the MeDi and the DASH diets. It recommends ingesting green leafy vegetables six times a week, other vegetables at least once a day, and two or more servings of berries per week. The MIND diet advocates snacking on nuts as well as consuming beans every other day, poultry twice a week, and fish at least once a week. Finally, the proposed diet sets very strict limits on butter, cheese, fried food, and fast food. A study among 960 participants over 4.7 years. Morris et al. conducted a study that implemented the dietary components from both the MeDi and the DASH diet shown to be neuroprotective, such as high consumption of vegetables and consumption of berries to develop the MIND diet. Those who adhered to the MIND diet the most tended to have a more favorable risk profile for preserving cognitive abilities including higher education, greater participation in cognitive and physical activities, and lower prevalence of cardiovascular conditions (103, 104).

Several common trends can be highlighted among these diets. They all recommend avoiding trans-unsaturated fatty acids (trans-fats) found in processed foods, such as partially hydrogenated oils, since they are known to raise the low-density lipoprotein (LDL) and decrease the high-density lipoprotein (LDL). Studies have shown that eating trans-fats increases the risk of developing heart disease, stroke, and type two diabetes; all conditions that can negatively affect brain health (105). Trans-fats are not only found in large amounts of processed foods, but also fried foods, baked goods, and a large variety of products found in super markets such as margarine, crackers, cookies, and pizzas.

Options for healthy dietary fats, on the other hand, include polyunsaturated fatty acids like omega-3 fatty acids. Given the vast amount of literature available on omega 3-fatty acids and brain health, it is important to highlight its beneficial qualities. Studies on omega-3 fatty acids have focused on the full range of fatty acids as opposed to examining the specific types. Docosahexaenoic acid (DHA) is the most prevalent omega-3 fatty acid in the brain and is involved in neuronal membrane plasticity (106). Although the most widely known source of omega-3 fatty acids is fish, there are plant sources that provide omega-3 fatty acids as well. These include olives, canola, flaxseed, soybeans, and butternut squash. Nuts and seeds such as walnuts and sunflower seeds also contain high levels of vital omega-3 fatty acids. Although plant sources of omega-3 fatty acids exist, the evidence supporting their beneficial properties is not as substantial as it is for fish (107).

The topic of salt has been the focus of many studies. There is well-established evidence supporting the connection between high sodium consumption and stroke (101, 108). The American Heart Association recommends that Americans lower their sodium intake to <1,500 mg per day because of the correlated health benefits. Research has shown that this guideline not only reduces the risk of stroke, but also decreases the risk of heart attack and heart failure (109).

Other topics of interest are the consumption of caffeine and coco flavonoids. Several studies have shown a significant relationship between coffee and tea consumption and decreased cognitive decline (110). These benefits have been attributed to polyphenol; a chemical compound found in plant-based food that is a vital antioxidant. The type and amount that must be consumed to be beneficial to brain health over an individual's lifespan have not been stipulated. However, a short-term benefit of coffee and tea consumption is increased alertness. Regarding cocoa flavonoids (often found in dark chocolate), there have been studies conducted researching the effects they have on brain health to enhance cognition (111, 112). Indeed, preliminary data suggest that cocoa flavonoids could have a positive effect on brain health; however, the study performed on participants with cognitive impairment involving cocoa flavonoids was inconclusive.

It is impossible to talk about nutrition without discussing the issue of weight. Several studies have linked obesity in an individual's midlife to an increased risk of future cognitive decline (113). Although, there is controversy over whether weight loss at an older age is productive. In order to effectively optimize your diet to improve your brain and heart health, nutritious, lower calorie options are the most beneficial. A study involving a 4-year active lifestyle intervention phase as well as a 9-year extended follow-up was conducted to investigate associations of long-term nutrient intake, physical activity, and obesity with later cognitive function (114). The results indicated that lower intake of total fat and saturated fatty acids and frequent physical activity were associated with better cognitive performance. Higher BMI and waist circumference were also associated with worse performance.

In conclusion, current research strongly suggests the beneficial effects of consuming polyunsaturated fatty acids (especially fish) and unprocessed, plant-based nutrients. Additionally, limits should be placed on the consumption of salt as well as on processed foods for they are rich in harmful fats such as LDLs and could be directly or indirectly harmful to brain health.

## The Brain and Social Connectedness

Humans are, in essence, social animals. Our growth, experiences, and some of the variances of our personalities are molded through social engagement. Specifically, the different levels of success in social engagement are major drivers of our quality of life (115). Typically, the goal of social engagement is to relate to others in positive ways through multi-factorial social networks. For the purpose of this discussion, the notion of social engagement refers to social interactions that have positive outcomes and are pleasing and meaningful to those who engage in them.

Unfortunately, the literature on this subject is limited because animal models of social engagement are designed and executed in highly controlled environments in which all possible relationships between the participants are closely monitored (116). This approach would not be ethical to apply in a human sample; therefore, studies that aim to observe cause and effect, also known as randomized controlled trials, have not been applied in this area of brain health. To gather evidence, researchers must rely mainly on epidemiological studies in the area of population biology. This approach assesses different levels of social interaction and compares individual experiences to each other instead of controlling levels of social engagement and determining the effects they have on brain health. It is important to note that many variables in real life become blended into social engagement situations. For example, people often have meaningful social interactions around a meal or specific sport activity; therefore, human studies on social engagement may not always be able to establish a causal relationship between brain health and social interaction. That being said, solid research suggests a possible impact of social engagement in brain health (115–120).

Notably, there are a few randomized controlled trials that tested the impact of real-world communal engagement in adults 50 and over. The two most substantial studies conducted are the Experience Corps (55, 121) and the Synapse Project (122). These studies were both randomized, controlled trials that exemplified various structures, functions, and qualities of social activities. The Experience Corps trial compared older adults that either volunteered to participate in the Experience Corps activities or who had the opportunity to join volunteer opportunities in the Baltimore area. The 702 randomized subjects of 60 years or older that were included in the study were evaluated at the 4-, 12-, and 22-month mark over a 2-year trial. Participants in the randomized Experience Corp group had significantly higher levels of generative desire and higher percentages of generative achievement than the controls at each follow-up point. Furthermore, the more hours of engagement in the Experience Corp program, the greater the benefit. A follow-up randomized, controlled study involving 111 men and woman in the same manner as the previous group was conducted to evaluate the impact of intervention on biomarkers of brain health. This subsequent study showed that participants in the Experience Corp had a significant increase in hippocampal volume. These results not only exemplify the clinical impact of intervention, but show a clear biological correlation between brain health and intervention (56).

The second study mentioned is the Synapse Project. This project had a similar hypothesis evaluating if the participation in group activities would aid in the maintenance of cognitive health in older adults. Those in the productive engagement groups were directed to spend 15 h per week in the Synapse environment, broken down into 5 h of formal instruction and 10 h of course assignments. There was a total of three productive engagement groups: a photo group, a quilting group, and a combined group. The control group involved an activity that mimicked a social club. Finally, there was a placebo group in which participants spent 15 h per week engaging in activities that utilized existing knowledge (i.e., watching TV and documentaries or reading magazines). The results indicated that a specific type of memory (episodic) significantly increased in subjects involved in the productive groups, supporting the hypothesis that sustained engagement could have benefits in brain health (122).

While strong social bonds seem to be a powerful element supporting brain health, loneliness caused by poorly functioning relationships, on the other hand, increases the risk of mental and social disorders. It is important to note that loneliness is not the same as isolation; people can feel lonely even when surrounded by others (123). Loneliness is the gap between the social engagement they want and what they currently have. Unfortunately, as people age, social networks tend to become more limited. They can begin to diminish as death, disease, or illness arise, enhancing the feeling of loneliness. Studies have shown that loneliness increases the risk of cognitive decline in older adults (124–127) significant loneliness being more detrimental than episodic loneliness. Of interest, some early research suggests that the effect of digital engagement on an individual's cognitive ability later in life is similar to that observed in in-person communication (128–130). In fact, a study conducted in the US revealed that older adults, after learning to use Facebook, performed 25% better in memory tests than non-Facebook users (131).

Another significant aspect of social connections are close relationships such as marriage. The impact of relationships like marriage have been studied from both the physical and emotional point of view. Participating in a fulfilling romantic relationship can be very beneficial (132, 133), however, relying exclusively on one primary relationship for social interactions can lead to isolation if something were to happen to the other individual involved in this primary relationship. Evidence shows that the loss of a spouse causes dysregulation of stress related hormones such as cortisol. Increased cortisol has been association with decreased hippocampus volume and decreased cognitive performance (134, 135), emphasizing the importance of developing a range of social relationships.

Additionally, research examining the benefits of human-animal interactions on human health is a continued source of interest. These human-animal relationships can be positive and meaningful (136–139), resulting in increased social contact and reductions in depression, anxiety, and social isolation. In fact, research has found that Alzheimer's disease patients have shown less verbal aggression and anxiety in the presence of animals. Similar positive effects can also be found in relationships with neighbors and other community entities (140–143). Higher neighborhood social cohesion provides residents with a feeling of belonging and elicits positive health behaviors such as increased physical activity and more smoking cessation attempts.

In conclusion, developing social connections is a fundamental need among humans that can enhance brain health and even improve memory. It is vital to engage in a range of social networks because relying on one may be detrimental and lead to loneliness. Whether the connection is shared between other humans or animals, online or in-person, social engagement drives our quality of life and feeling of belonging.

## Author Contributions

JM drafted the manuscript and critically reviewed the manuscript. KD and AK assisted drafting and editing the manuscript. SL, LC, and NB reviewed the manuscript and provided comments. All authors will approve the final version of the manuscript and agree to be accountable for the content of the work.

### Conflict of Interest

The authors declare that the research was conducted in the absence of any commercial or financial relationships that could be construed as a potential conflict of interest.
